# Base Excision Repair: Mechanisms and Impact in Biology, Disease, and Medicine

**DOI:** 10.3390/ijms241814186

**Published:** 2023-09-16

**Authors:** Dhara Gohil, Altaf H. Sarker, Rabindra Roy

**Affiliations:** 1 Department of Oncology, Lombardi Comprehensive Cancer Center, Georgetown University, Washington, DC 20057, USA; dg1061@georgetown.edu; 2 Lawrence Berkeley National Laboratory, Berkeley, CA 94720, USA; ahsarker@lbl.gov

**Keywords:** base excision repair (BER), 5′-Gap, nucleotide incision repair (NIR), transcription-associated, replication-associated, RECQ1, XPF-ERCC1, OGG1, NEIL1/2, PNKP, APE1, PARP, POL β, XRCC1

## Abstract

Base excision repair (BER) corrects forms of oxidative, deamination, alkylation, and abasic single-base damage that appear to have minimal effects on the helix. Since its discovery in 1974, the field has grown in several facets: mechanisms, biology and physiology, understanding deficiencies and human disease, and using BER genes as potential inhibitory targets to develop therapeutics. Within its segregation of short nucleotide (SN-) and long patch (LP-), there are currently six known global mechanisms, with emerging work in transcription- and replication-associated BER. Knockouts (KOs) of BER genes in mouse models showed that single glycosylase knockout had minimal phenotypic impact, but the effects were clearly seen in double knockouts. However, KOs of downstream enzymes showed critical impact on the health and survival of mice. BER gene deficiency contributes to cancer, inflammation, aging, and neurodegenerative disorders. Medicinal targets are being developed for single or combinatorial therapies, but only PARP and APE1 have yet to reach the clinical stage.

## 1. Introduction

### 1.1. DNA Damage

DNA damage can arise from either endogenous or exogenous sources [1]. Most endogenous damage stems from biochemical reactions such as DNA mismatches due to errors in replication and repair. In addition, topoisomerase–DNA complexes can lead to DNA misalignment or generate suicidal complexes when irreversibly trapped in DNA lesions. Other sources include spontaneous base deamination, abasic sites, and DNA methylation. Oxidative stress, the imbalance between the toxicant’s ability to produce reactive oxygen species (ROS) and the body’s ability to detoxify them [2], can result in mismatch base pairing or unpairing.

Conversely, exogenous means include external factors such as ionizing and ultraviolet radiation, chemicals such as alkylating agents and aromatic amines, carcinogens in the air, natural dietary carcinogens, food pyrolysis products, exposure at the workplace, voluntary exposure, and therapeutic drugs [1,3]. Exogenous agents can damage DNA directly or require metabolic activation to react with DNA constituents [4].

### 1.2. Background

Due to the various sources of DNA damage, the body must have a robust repair system. Most DNA damage can be segregated as single-strand breaks (SSBs) or double-strand breaks (DSBs), each with its own repair mechanisms. DSBs are primarily repaired through either non-homologous end joining (NHEJ) or homologous recombination (HR). SSBs generated directly in DNA corrected by single-strand break repair (SSBR). SSBs arising as repair intermediates are either repaired through mismatch repair (MMR) for base mismatches during replication and insertion–deletion loops [5], nucleotide excision repair (NER) for removing bulky lesions, or base excision repair (BER) for base damage that appears to have minimal effects on the helix [1].

Despite the repair mechanism needed, the initial response starts with the DNA damage response (DDR) pathways. This process involves recognizing the DNA lesion and initiating the signaling cascade for repair [6]. DDR is activated by one of three phosphoinositide 3-kinase (PI3K)-related kinases (PIKKs). DNA-dependent protein kinase (DNA-PK) binds to DSBs, primarily for NHEJ repair. Ataxia-telangiectasia mutated (ATM) is recruited to chromatin to promote HR but also has a minor role in NHEJ. ATM- and Rad3-related (ATR) are commonly found in proliferating cells and respond to a broader range of genotoxic stresses. All three kinases, DNA-PK, ATM, and ATR, are regulated by specific protein co-factors—Ku80, NSB1, and ATRIP, respectively. Currently, no known DDR pathway directly influences BER. However, in the G1 phase, once the base damage gets converted to an SSB, the nick/gap can be detected by ATR. During the S phase, ATR is activated when base damage is found in stalled forks or gaps generated during replication. Lastly, in the G2/M phase, the gaps can become nick/gaps or catenanes [7].

**Figure 1:**
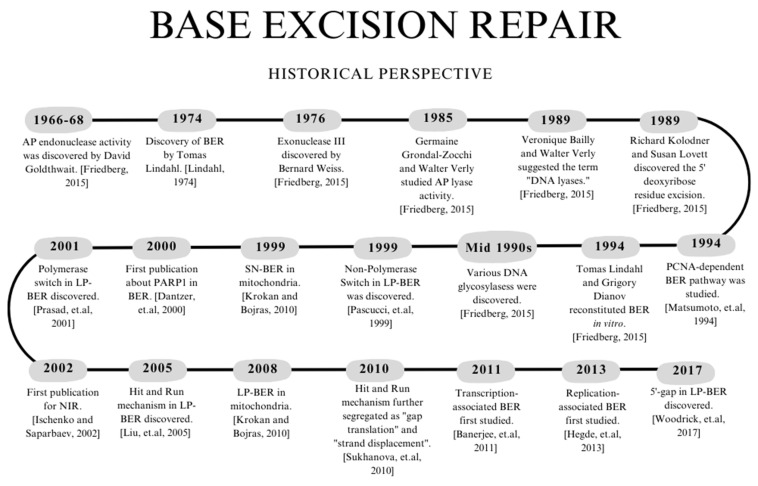
Timeline highlighting the discovery of various BER enzymes and pathways. References for the events in the figure are as follows: Lindahl, 1974 [8]; Friedberg, 2015 [9]; Krokan and Bjoras 2013, [10]; Pascucci et. al., 1999 [11]; Matsumoto et. al., 1994 [12]; Dantzer et. al., 2000 [13]; Woodrick et. al., 2017 [15]; Prasad et. al., 2001 [16]; Ischenko and Saparbaev, 2002 [17]; Liu et. al., 2005 [18]; Sukhanova et. al., 2010 [19]; Banerjee et. al., 2011 [20]; Hegde et. al., 2013 [21]. The figure was generated using Canva.com (accessed on 15 August 2023).

BER was first studied in 1974 by Tomas Lindahl when he discovered uracil-DNA glycosylase (Ung) in *Escherichia coli*. Lindahl reasoned that the abasic site would interact with an AP endonuclease, a DNA polymerase, and a ligase, thus highlighting the major components of BER without specifying any proteins [8]. In 1994, Lindahl and Grigory Dianov reconstituted BER in vitro using Ung [9]. During the mid-1990s, various DNA glycosylases were discovered. Before the turn of the century, it was thought that mitochondrial DNA could only perform SN-BER [10]. However, in 2008, three individual research labs showed that LP-BER also occurs in mitochondria. In 1999, Non-Polymerase Switch was the first LP-BER sub-pathway discovered [11] (mechanism details are in Section “Non-Polymerase Switch”). While not officially recognized as LP-BER, Matsumoto et al. showed PCNA-dependent BER in 1994 [12]. In 2000, it was demonstrated that PARP1 is needed for BER using both in vitro and in vivo studies [13]. Before this, researchers showed that PARP1 is required for SSBR induced by gamma radiation [14]. However, this work does not directly connect to BER since SSBR occurs downstream. In addition, the type of damage induced generates SSBs that are not equivalent to the SSBs found in BER. In 2017, the 5′-Gap LP-BER sub-pathway was discovered [15] (mechanism details are in Section “5′-Gap”). Figure 1 highlights essential events related to discovering various BER enzymes and pathways. Ongoing work in the field is related to understanding genome instability arising from the dysregulation of different BER proteins and their connection(s) to certain diseases as well as identifying various BER targets for therapeutics.

Through transcription-coupled nucleotide excision repair (TC-NER), Philip Hanawalt’s lab first showed the repair of bulky lesions induced by UV damage from the transcribed strand of the active gene. However, these lesions could not be repaired in the cells of individuals with Cockayne syndrome (CS), which suggested that the lack of repair results in CS [22,23]. However, CS is not only caused by the defects of *CSA* or *CSB* gene products, but also by the defects of genes involved in repairing UV lesions by NER, such as *XPG*, *XPF*, and *XPB*. The *XPG* gene product is interesting because only the loss of XPG protein but not its catalytic mutant results in the onset of CS phenotypes, arguing lesions other than UV repair defects may result in CS [24]. Studying oxidative DNA lesions led to understanding transcription-coupled base excision repair (TC-BER) [25]. CS (CSA and CSB) cells were sensitized to agents that induce oxidative DNA damage, such as hydrogen peroxide, potassium bromate, and γ-rays [26]. In addition, a transcription-dependent role of CSB in the repair of oxidative lesions was reported [27]. These suggested the involvement of BER-mediated repair linked to transcription.

## 2. Base Excision Repair (BER) Mechanisms

### 2.1. Global

**Figure 2:**
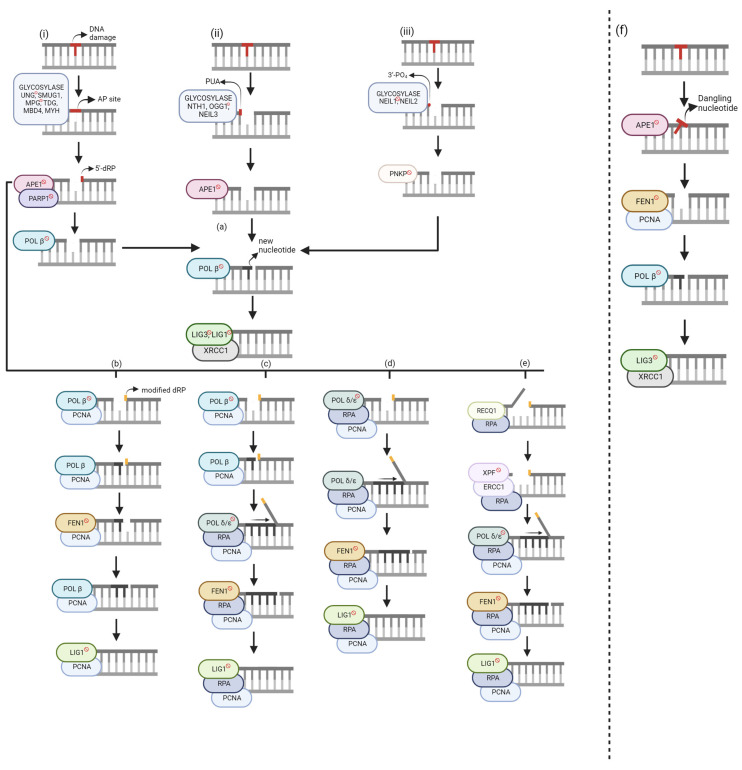
Global base excision repair (BER) mechanisms. Unlabeled DNA ends are either polymerase extendable clean 3′-OH groups or 5′-PO_4_ groups that can be ligated. (**i**) Monofunctional glycosylase. (**ii**) Bifunctional glycosylase—β elimination. (**iii**) Bifunctional glycosylase—β, δ-elimination. (**a**) Short-Nucleotide BER (SN-BER). (**b**–**e**) Long-Patch BER (LP-BER). (**b**) Hit and Run. (**c**) Polymerase Switch. (**d**) Non-Polymerase Switch. (**e**) 5′-Gap. (**f**) Short-Patch Nucleotide Incision Repair (NIR). Genes with [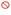] sign are target BER inhibitors. The figure was generated using Biorender.com (accessed on 20 August 2023).

Global repair mechanisms can be applied to any section of the genome. Specifically, base excision repair (BER) corrects forms of oxidative, deamination, alkylation, and abasic single-base damage that appear insignificant to the helix [1]. Oxidative damage, from either metabolic or environmental sources, can generate ROS, affecting both the DNA strand and dNTP pool. Oxidized bases can lead to abasic sites, and accumulation of such damage can result in mutagenesis and cell death [28]. Damage within the dNTP pool can result in the incorporation of oxidized bases during repair or replication, ultimately leading to ligation failure. Currently, three categories of DNA glycosylases recognize damaged bases and there are two repair classifications—short-nucleotide (SN-BER) or long-patch (LP-BER). Within global BER, nucleotide incision repair (NIR) is an alternate pathway. Figure 2 categorizes the DNA damage based on the DNA glycosylase-mediated repair intermediates and the six known repair pathways.

Despite the BER pathway, various proteins are coordinated through transient protein–protein interactions or constitutive stable repair complexes [29]. The “passing the baton” model states that the repair intermediates are passed from one protein to the next until ligation is complete. However, its efficiency is limited to SN-BER. Another model suggests that the repair proteins are preassembled in a repair complex, as shown through co-immunoprecipitation experiments. Although there is no evidence of a stable complex containing all BER proteins in a single complex [29], research has shown that in addition to pre-existing sub-complexes, certain protein–protein interactions occur at the damage site [30,31,32,33,34]. In addition, glycosylase and APE1 may work independently of these complexes. Furthermore, it has been proposed that scaffolding accessory proteins may be needed for BER complex formation [35]. While it has no known catalytic function and is not required for in vitro BER reconstitution experiments, scaffolding proteins are needed for BER in vivo.

For efficient repair, the gene expression of every repair protein must be at its optimal level, which is assumed when discussing the various mechanisms in this section. The balance between SN- and LP-BER depends on the relative concentration of BER enzymes and scaffolding proteins and persistent 5′ blocking lesions at the repair site [36]. Genome instability, caused by over or under-expression of certain proteins, can lead to various deficiencies and diseases. An inefficient repair can lead to the accumulation of toxic lesions and, ultimately, apoptosis. For example, although base excision using a glycosylase is the first step in BER, higher amounts of these repair intermediates are more toxic than the parental damage if there are an insufficient amount of downstream repair proteins [37]. In addition, if a replication fork encounters a BER intermediate, the fork could collapse, resulting in a double-strand break [38], which can cause cell death if not repaired.

BER starts with a glycosylase excising the DNA base damage on the strand by hydrolyzing the *N*-glycosidic bond between the base and deoxyribose [39]. There are eleven known glycosylases, each recognizing specific DNA base lesions with overlapping specificities [10]. The choice of glycosylase for the same lesion may depend on the cellular state and cell types [40]. Glycosylases are categorized as either monofunctional or bifunctional. Monofunctional glycosylases (Figure 2i) only possess glycosylase activity and generate a hydrolytic, non-coding apurinic/apyrimidinic site (AP site) [5]. Conversely, bifunctional glycosylases have an additional function—3′-AP lyase activity. There are two groups of bifunctional glycosylases, which are segregated based on the type of reaction used to excise the DNA substrate, β-elimination (Figure 2ii) or β,δ-elimination (Figure 2iii), resulting in either 3′-phospho-α, β-unsaturated aldehyde (PUA), or a 3′-PO_4_, respectively.

AP sites and 3′-PUAs can be removed using human apurinic/apyrimidinic endonuclease 1 (APE1) but yield different products. APE1 works with poly-(ADP-ribose) polymerase 1 (PARP1) to remove the AP site by cleaving at the 5′ site, resulting in a 5′-deoxyribose phosphate (5′-dRP). When bound to DNA, PARP1 catalyzes poly(ADP-ribose) synthesis, allowing nuclear protein covalent modification. Conversely, autopoly(ADP-ribosyl)ation decreases DNA-binding activity and thus its dissociation from DNA. Although the mechanistic role of PARP1 is not entirely understood in BER, some functions have been explained. During POL β-mediated LP-BER stimulated by either APE1 or FEN1, PARP1 inhibited this pathway, but its effects were diminished once ribosylated [19]. There was no effect of PARP1 in SN-BER, impact on repair efficiency, or recruitment of BER proteins [29]. However, it protects single-strand breaks (SSBs) from nucleases or prevents the conversion to more toxic damage by dimerizing if the molar amount of SSBs exceeds the molar amount of needed BER enzymes [29]. APE1 removes the 3′-PUA, whereas polynucleotide kinase 3’-phosphatase (PNKP) excises 3′-PO_4_ groups, resulting in unmodified 3′ and 5′ ends. At this stage, either short-nucleotide or long-patch BER is chosen.

#### 2.1.1. Short-Nucleotide BER (SN-BER)

Both monofunctional and bifunctional glycosylases can participate in SN-BER (Figure 2a). After a monofunctional glycosylase, APE1 generates a 5′-dRP, lysed by POL β’s lyase activity, resulting in unmodified 3′ and 5′ ends. From this point, all three glycosylase pathways have resulted in clean ends. Therefore, POL β can incorporate the correct nucleotide. The nick is sealed by DNA Ligase 3 (LIG3), with X-ray repair cross-complementing protein 1 (XRCC1) assisting. XRCC1 is a scaffold protein associated with POL β, LIG3, and human polynucleotide kinase (PNK), but its biochemical role is unknown [34]. Mutational analysis and binding studies have shown that LIG1 interacts with PCNA, and LIG3 interacts with the BRCT domain of XRCC1 [41]. Further research showed that LIG1 is applied to both SN-BER and LP-BER in nuclear DNA, while LIG3 repairs mitochondrial DNA. Still, it emphasized that further research must consider different base lesions, cell types, and other mammalian species [10]. Large amounts of damage to the dNTP pool can result in POL β incorporating an oxidized base, ultimately causing ligation failure [28]. The base correction function of BER is strongly backed up by trans-lesion DNA synthesis (TLS) polymerases; however, they often cause misincorporation and mutations [29].

#### 2.1.2. Long-Patch BER (LP-BER)

Although SN-BER can be applied to any of the three pathways, LP-BER starts with a modified 5′-dRP. If the sugar group is either oxidized or reduced, POL β lyase activity cannot function, resulting in long-patch BER. Currently, there are four published LP-BER mechanisms with several commonalities. The primary differentiation factor between these sub-pathways is/are the polymerase(s) used for incorporating the new nucleotides.

##### Hit and Run

Although POL β cannot use its lyase activity to excise the modified 5′-dRP, a new mechanism within LP-BER showed that POL β and FEN1 work together using single-nucleotide gaps [18] (Figure 2b). The researchers showed that POL β had an increased binding affinity to the DNA substrate with excess FEN1 expression. This sub-pathway was discovered because it was revealed that although flaps with a 5′-terminal tetrahydrofuran (THF) residue were poor substrates for POL β-mediated repair, FEN1 stimulated DNA synthesis via POL β. In addition, PARP1 promoted POL β for LP-BER synthesis, but only in the presence of FEN1. However, the mechanism for this interaction is not known.

The researchers explained an alternating catalytic cycle between POL β and FEN1 for this sub-pathway. POL β incorporates one nucleotide, but the nicked flap remains, which is said to be the rate-limiting step. FEN1 cleaves the nicked flap and an additional nucleotide, creating a substrate for POL β to add another nucleotide via gap translation. While there would be no extra flaps, this alternating cycle would continue until ligation. This pathway needs proliferating cell nuclear antigen (PCNA) to localize the proteins to the DNA substrate and stimulate FEN1 activity [42,43]. Although initial research showed that this mechanism could be applied for a 2–11 nucleotide gap [18], further study segregated POL β-mediated LP-BER [19]. Gap translation can be used for 2-nucleotide repair. Alternatively, POL β can perform strand displacement when incorporating new nucleotides, allowing for an 11-nucleotide repair when stimulated by APE1 due to its 3′ to 5′ exonuclease activity [19]. POL β can perform strand displacement at high protein concentrations or when stimulated by specific protein–protein interactions [5]. Long-patch repair mechanisms use Ligase 1 (LIG1) rather than Ligase 3 (LIG3).

##### Polymerase Switch

POL β is involved in short-nucleotide and long-patch repair, but its highest catalytic activity is on 5′-phosphorylated 1-nucleotide (nt) gapped DNA [44]. Therefore, in this sub-pathway, POL β has been shown to incorporate the first nucleotide in the repair gap [45] (Figure 2c). POL δ/ε incorporates the remaining nucleotides to form the 2–11 nucleotide patch. POL δ/ε has 3′ to 5′ exonuclease activity, which is useful for proofreading errors created by proofreading-defective POL δ/ε molecules [46]. Whereas FEN1 cleaved 1-nt at a time in the Hit-and-Run mechanism, the 2–11 nt flap is now cleaved at once. Human replication protein A (RPA) stimulates FEN1 and LIG1 activity and binds to ssDNA for transient stability [47]. At the 5′ terminus, RPA binding allows for repair completion. Alternatively, RPA may bind at the 3′ end of the upstream primer to allow the DNA to remain unwound until POL ε can complete its proofreading function.

##### Non-Polymerase Switch

Unlike the previous sub-pathway, only POL δ/ε is needed for new nucleotide incorporation (Figure 2d). Although these polymerases can complete a 1-nt repair, the repair intermediate is inefficiently ligated [11] because a 1-nt incorporation does not generate strand displacement. Therefore, FEN1 would not be able to cleave the modified 5′-dRP. Downstream steps are like the Polymerase Switch sub-pathway.

##### 5′-Gap

In 2017, an LP-BER sub-pathway was discovered that creates a 9-nt gap on the 5′ side (Figure 2e) [15]. RECQ1, a DNA helicase, unwinds the DNA and due to its 3′–5′ activity, creates an 8-nt long 5′-flap, which is excised by ERCC1-XPF – a heterodimeric endonuclease that catalyzes a 5′ incision. A 9-nt gap is formed on the 5′ side of the lesion.

The RECQ1-PARP1 relationship regulates 5′ gap formation, suggesting that RECQ1 may be a PARP1 biological inhibitor. In addition, in the presence of PARP1 (independent of its poly ADP-ribosylation activity), RECQ1 significantly increased its binding to the substrate. Therefore, RECQ1 requires PARP1 to initiate this sub-pathway but inhibits PARP1’s activation.

Once the 9-nt gap is formed, POL δ/ε adds the appropriate nucleotides. PCNA, FEN1, and RPA are needed for similar functions as the other LP-BER pathways. RPA may also stimulate RECQ1. Ligation may be completed using either LIG1 or LIG3; however, the ligase type has not been identified. From the initial damage site, twenty nucleotides are incorporated.

Although the pathway was first discovered using plasmid-based studies, experiments conducted using genomic DNA showed this pathway can be applied to repair initiated by either monofunctional or bifunctional glycosylases and validated RECQ1-PARP1 regulation. Possible advantages of this pathway would be allowing for repair in PNKP-deficient mutants or repair after 8-oxodG incorporation by POL β/λ in high oxidative stress conditions. Although POL δ/ε has 3′ to 5′ proofreading capabilities, it cannot correct 8-oxodG lesions since the bypass efficiency is 60–70% [48] and cannot correct the 3′-terminal 8-oxodG [46]. APE1 and TDP1 may remove these 3′ modifications but have only been tested in in vitro experiments [28].

#### 2.1.3. Nucleotide Incision Repair (NIR)

In 2002, Ischenko and Saparbaev discovered a glycosylase-independent repair pathway, which they named nucleotide incision repair (Figure 2f) [17]. APE1 creates an incision at the damage site by hydrolyzing the phosphodiester bond. This results in a damaged deoxynucleotide with 5′-PO_4_ and 3′-OH groups [49]. FEN1 and PCNA cleave the dangling nucleotide. Like SN-BER, POL β incorporates the new nucleotide, and LIG3/XRCC1 seals the nick. Advantages of this pathway include that it avoids the formation of toxic intermediates and explains DNA repair proficiency in glycosylase-deficient mutants [17]. However, it is crucial to consider that glycosylases may have a stronger binding affinity to the DNA substrate than APE1, making BER the preferred pathway. In addition, it is evolutionarily conserved, supporting the existence of a back-up repair pathway in organisms. Lastly, it may serve as a new physiological target for LP-BER but is currently not confirmed. It has been suggested that BER and NIR work together to provide genome stability since the rate of cleaving a dihydrouridine-containing (DHU) substrate by APE1 in NIR is comparable to the rate of cleaving an AP site in BER [49].

Although glycosylases (in BER) and APE1 (in NIR) both recognize damaged DNA, they have different structural DNA-binding sites and different amino acid residues that are involved in recognizing the damaged nucleotide [50]. APE1 substrate specificity factors the enzyme bending the damaged DNA, the damaged nucleotide turning inside out from the helix, and having specific contact between the base and the enzyme’s active site. In addition, GC-rich regions are preferred targets for BER, but do not all contain regions for Apn1-binding [51].

### 2.2. Transcription-Associated

DNA damage significantly threatens DNA replication and transcription if left unrepaired or repaired improperly. Since lesions placed on the non-transcribed strand have little or no effect on elongating RNA polymerase II (RNAPIIo—the hyper phosphorylated form of the polymerase) transcription, all cases discussed place the lesion on the transcribed strand. During transcription, RNAPIIo often encounters DNA lesions in the transcribed strand, causing RNAPIIo to pause or stall at the sites of the lesion; this could be the source of transcription-stress and mutagenesis, leading to varieties of disease phenotypes including aging and age-related pathologies. In normal cells, transcription-blocking lesions could be repaired by the conserved transcription-coupled repair (TCR) pathway that preferentially repairs DNA lesions from the transcribed strand of the active gene. UV irradiation or other genotoxic stressors block RNAPIIo. In mammalian cells, this signals TCR to repair bulky DNA lesions [52]. Several recent publications suggest the existence of a dedicated TCR pathway to repair oxidative DNA lesions [27,53,54].

#### 2.2.1. Apurinic/Apyrimidinic (AP) Sites

AP sites are the most frequently generated lesions in the cell (approximately 10,000 per cell per day), predominantly caused by spontaneous hydrolysis of the glycosidic bond between deoxyribose and nucleobase or the damaged base removed by the DNA glycosylase (Figure 2) [55]. In vitro assays show that mammalian RNAPIIo assists AP site repair by blocking transcription on the transcribed strand [54].

#### 2.2.2. Pyrimidine Modifications

Pyrimidines, such as cytosine modification products and their oxidized derivatives, are common epigenetic modifications that have a limited impact on transcription in eukaryotes. RNAPIIo is either bypassed or slightly paused during elongation and only slightly compromises transcription fidelity. Certain thymine modifications caused by components of cigarette smoke strongly impede transcription, suggesting that the position of modifications has an impact on transcription [56]. Uridine that arises in DNA because of cytosine deamination does not stall transcription, but RNAPIIo incorporates adenine opposite uracil during transcription. Template strand containing multiple uridines is transcribed but with low fidelity, possibly due to misincorporation. Two other oxidized pyrimidine lesions, 5′-OH cytosine and thymidine glycol, are bypassed by RNAPIIo with slight pause [57].

#### 2.2.3. Purine Modifications

Purine oxidation generates a wide range of products; 8-oxoguanine (8-oxodG) is the most abundant, which can be further oxidized to non-bulky hydantoin lesions, such as guanidinohydantoin (Gh) and spiroiminodihydantoin (Sp) [58]. RNAPIIo bypasses 8-oxodG lesions predominantly with misincorporation of adenine [54,59,60], but the oxidation products of 8-oxodG block RNAPIIo and favor misincorporation of purines [61,62]. Oxidation of a purine nucleotide by an OH radical can result in a crosslink between the nucleobase and deoxyribose, leading to the formation of CydA and CydG; both have high effects on transcription by causing RNAPIIo stalling [63]. Although uracil and cytosine are incorporated opposite CydA and CydG, respectively, adenine is incorporated in the following position, independent of the template. Consequently, this causes structural changes that make it difficult for RNAPIIo to recognize the 3′ end for continuous extension.

#### 2.2.4. Single-Strand Breaks

Most DNA lesions in the cells (~75%) are SSBs arising from oxidative damage during metabolism or processing of AP sites. A nick in the template strand with damaged termini inhibits RNAP synthesis. If SSBs are not repaired rapidly and accurately, they can lead to cell death, chromosomal aberrations, and genetic mutations [64,65].

#### 2.2.5. Evidence of BER-Linked Transcription in Mammalian Cells

Accurate repair of oxidative lesions is essential from the transcribing region to avoid the generation of mutant transcripts. However, most oxidized DNA bases only pause RNAPIIo elongation at the expense of transcriptional mutagenesis [66,67,68]. It is known that guanine is highly vulnerable to oxidation due to having a low redox potential, causing the formation of abundant 8-oxodG lesions, which only cause minor helix distortions, allowing RNAPIIo to bypass it unless processed by its specific glycosylase OGG1 [69].

NEIL2 is a particularly important glycosylase for the repair of damages linked to transcription because of its interaction with RNAPIIo, transcription–repair coupling factor CSB, and transcription factor IIH (TFIIH) [20,70]. In addition, it prefers lesions with the bubble structure that form during transcription. NEIL2 immunocomplex containing RNAPIIo and hnRNPU (RNA splicing factor) have been shown to repair oxidized base lesions from the transcribed strand [20]. Studies have shown that with age, DNA damage was accumulated in the actively transcribed genes in *NEIL2*-KD cells and oxidized base lesions in the transcribed genes in *NEIL2* KO mice [71]. In addition, *NEIL2* expression is independent of the cell cycle. Cumulatively, this suggests that NEIL2 is involved in BER linked to transcription.

**Figure 3:**
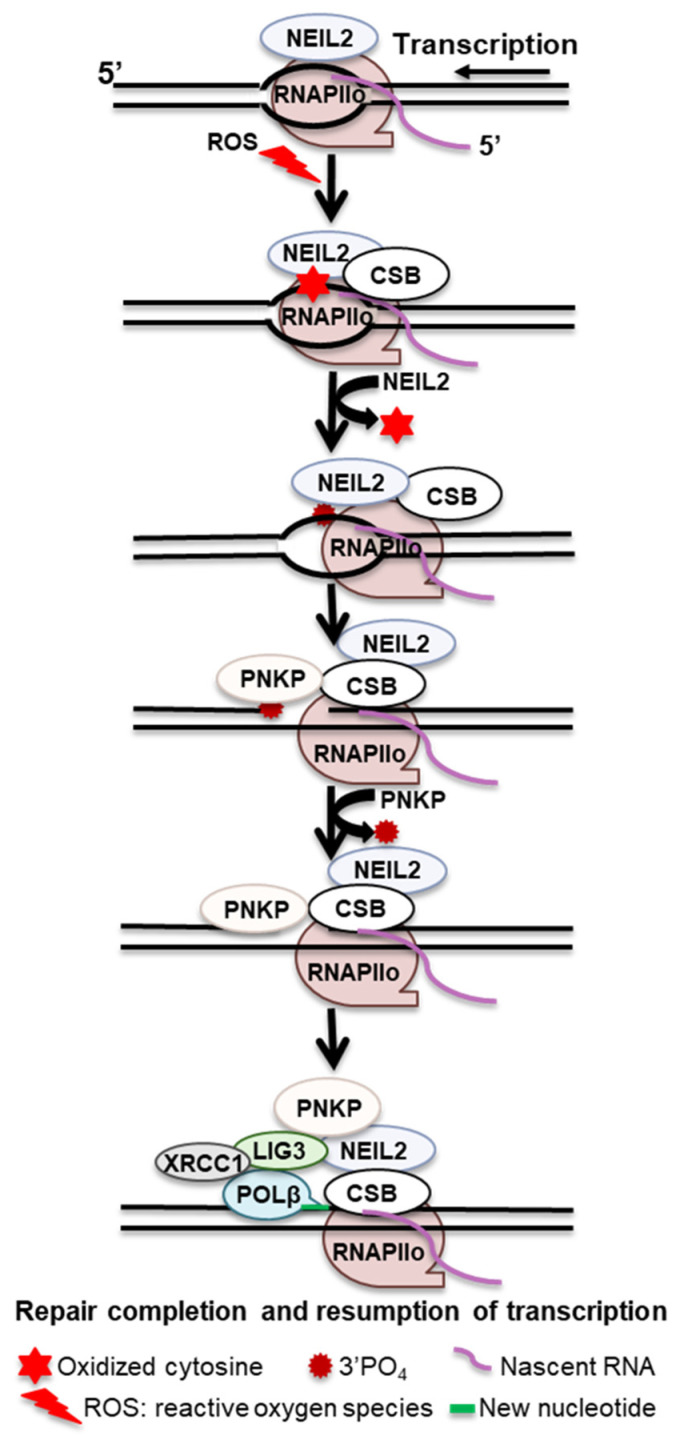
Proposed transcription-associated BER mechanism.

NEIL2 is a bifunctional glycosylase that incises the associated AP sites through its AP lyase activity. During TET-TDG-mediated active demethylation, after TDG cleaves the *N*-glycosidic bond, NEIL2 serves only to process the AP site, suggesting that in certain contexts, NEIL2 preferentially processes AP sites and may substitute for APE1 [72]. A proposed model demonstrates that when RNAPIIo encounters AP sites or oxidative DNA lesions (Sp and Gh) during transcription, RNAPIIo stalls and recruits CSB and TFIIH to the repair sites. CSB brings NEIL2 through protein–protein interaction and stimulates NEIL2 activity, generating single-strand breaks with 3′-phosphate termini, which further stalls RNAPIIo. Retrograde movement or remodeling of the stalled RNAPIIo facilitates PNKP to replace the 3′-PO_4_ for the 3′-OH end; POL β then inserts the missing base, and LIG3-XRCC1 seals the nick and transcription is resumed (Figure 3).

CSB and transcription-independent recruitment of OGG1 to 8-oxodG sites have been reported. However, recruiting CSB and XRCC1 to 8-oxodG sites depends on transcription, suggesting a transcription-dependent role of CSB beyond initial damage recognition [27]. Overall, CSB recognizes stalled RNAPIIo at OGG1-generated BER intermediates through its bifunctional glycosylase activity and recruits XRCC1 to mediate the coordination of subsequent repair factors for completion of SSB repair in transcribed genes.

### 2.3. Replication-Associated

Replication-associated repair refers to mechanisms that repair damaged DNA in coordination with the replisome. Some oxidized base lesions and abasic sites are potent blocks to replicative polymerases. The repair system does not always remove them before a DNA replication fork arrives. However, most oxidized lesions would not stall replicative polymerase but would cause mutations by inaccurate base pair during replication. Therefore, mutagenic base lesions must be repaired prior to replication to prevent mutation fixation. NEIL1 can repair oxidized lesions from structures mimicking the replication fork. In addition, its induced expression in the S phase of the cell cycle and stable physical and functional association with several proteins in the DNA replication complex cumulatively suggest that NEIL1 is part of the replication complex for repairing oxidized bases in the replicating template [21]. A model has been proposed that during replication, nonproductive NEIL1 binds to an oxidized lesion, such as 5-OHU or 5-OHC, in RPA-coated ssDNA; this stalls and regresses the fork that brings the lesion in the re-annealed duplex for faithful repair. The direct interaction of NEIL1 with RPA prevents DSB formation. NEIL1 repairs the lesion in association with replication machinery, including PCNA, RF-C, FEN1, POL δ, and LIG1; some are reported to stimulate NEIL1’s activity [73]. The functional association of NEIL1 with WRN or SMARCAL1 (both have ATPase activity) may coordinate fork regression and resolution to avoid fork collapse. Once repair is completed, replication resumes [74].

Recently, NEIL3 was shown to be induced during the S phase [75]; however, its substrate specificity has not been characterized extensively. NEIL3 may also be involved in the pre-replicative repair of different or overlapping substrates.

## 3. Biology and Physiology

Despite the various global BER mechanisms, it is not entirely understood how cells choose a particular repair pathway [76]. It has been shown that LP-BER is prevalent in cells with low ATP concentrations because poly(ADP-ribose) is stimulated [76,77]. In addition, long-patch BER is needed when POL β cannot remove the 5′-dRP. Lastly, long-patch is preferred when cells are in the S phase because more replication-associated proteins are abundant, and these proteins also have a function in BER, but 8-oxodG repair may be limited in non-replicating tissues [78]. Conversely, SN-BER is more prevalent in terminally differentiated cells and is the highest in spermatogenic germ cells [78,79]. Many repair proteins are evolutionarily conserved, supporting that BER is necessary for genome stability.

### 3.1. Gene Knockout Studies in Mouse Models

As highlighted when discussing mechanisms, each sub-pathway involves the coordination of various repair proteins. Studies conducted by multiple researchers showed the implications of specific gene knockouts in mouse models.

#### 3.1.1. Monofunctional Glycosylases

Single knockouts (KOs) of DNA glycosylases do not show immediate phenotypic changes, even though unrepaired base lesions have mutagenic effects. Starting with monofunctional glycosylases, *UNG* KO was the first example of cancer development without a carcinogen and increased incidence of B-cell lymphomas in old age [80]. SMUG1 was shown to be an essential glycosylase to modulate fat metabolism [81]. *SMUG1* KO in one-year-old mice increased weight and fat content and changed activity in enzymes needed for fatty acid regulation. Liver cells showed an accumulation of free fatty acids and triglycerides. *SMUG1* KO had additional DNA damage at telomeres in older mice and increased hepatocyte senescence. *AAG* (or *MPG*) KO showed some residual repair, but adding alkylating agents increased mutagenesis in splenic lymphocytes [82,83]. AAG is a unique glycosylase because it can identify various DNA substrates but cannot excise the lesion [37]. In some cell types, loss of function can increase its sensitivity to alkylating agents. There is no general trend regarding response to alkylating agents based on cell type. However, higher levels of *AAG* showed longer relapse-free survival in breast and ovarian cancer patients treated with CMF (cyclophosphamide, methotrexate, and fluorouracil).

TDG is involved in embryonic development. KO in mouse embryonic fibroblasts generated epigenetic changes that affected developmental genes [84]. *TDG* KO showed increased incidences of hepatocellular carcinoma and hepatoblastoma in male mice [85]. Furthermore, there was a two-fold increase in small-intestinal adenomas due to *TDG* KO [86]. Rather than functioning as a tumor suppressor, *TDG* KO inhibited the growth of colorectal cancer when xenografted on nude mice, and *TDG* expression was upregulated in human colorectal cancer [86]. In humans, *TDG* knockdown reduced the viability of human melanoma cell lines [87], but a mutation causing reduced expression increased incidences of rectal cancer [88]. These studies showed that *TDG* may act as a tumor suppressor or oncogene, depending on species and cell type. *MBD4* KO mice showed a higher mortality rate in response to chronic inflammation [89]. Lastly, *MYH* KO showed no direct correlation between age and accumulation of 8-oxodG but did show liver damage buildup. However, bi-allelic mutations have been shown to cause a predisposition to multiple colorectal adenomas and carcinomas [90].

#### 3.1.2. Bifunctional Glycosylases

Bifunctional glycosylases that catalyze β elimination are unique regarding the cell types affected but show a similar trend of no significant phenotypic changes. *NTH1* KO allowed for repair but was less efficient, supporting that glycosylase function may have some overlap regarding DNA substrates [91,92]. KO of *OGG1* showed a buildup of 8-oxodG in hepatocytes, with and without exposure to potassium bromate [93,94,95]. When exposed to UVB light, skin cells showed an increased tumor response [96]. *NEIL3* was expressed in the regenerative subregions of the brain and has a specialized function in proliferating cells. After hypoxia-ischemia, KO showed reduced microglia and loss of proliferating neuronal progenitors in the striatum [97].

Bifunctional glycosylase may also catalyze β, δ-elimination. NEIL1 is vital in preventing diseases associated with metabolic syndrome [98]. Heterozygotic and KO mice showed a combination of symptoms: obesity, dyslipidemia, fatty liver disease, and may develop hyperinsulinemia. mtDNA showed increased levels of DNA damage. In addition, the frequency and size of hepatocellular carcinomas were more significant in *NEIL1* KO mice [98]. *NEIL2* deficiencies are linked to diseases related to genome instability and/or inflammation [71]. KO showed telomere loss in embryonic fibroblasts and higher sensitivity to inflammatory agents such as lipopolysaccharide (LPS). Since single KO of glycosylases did not show phenotypic changes, it suggests that the single glycosylase KOs not only allow other glycosylases to function as back-up but also allow for NIR, as required, which further supports that BER and NIR work together to provide genome stability.

#### 3.1.3. Double Knockout Studies for Glycosylases

Some research has been conducted using double-knockout mouse models to study DNA glycosylases further. A double *OGG1/MYH* KO showed a heightened chance of tumors in the lung, lymphoma, ovary, and small intestine [99]. *NTH1/NEIL1* KO highlighted that the mice developed pulmonary and hepatocellular tumors at a much higher incidence rate than single knockout [100]. The *SMUG1/UNG* KO showed similar breeding patterns and health compared to the *SMUG1* KO, but the double knockout exacerbated cancer predisposition in *MSH2* KO mice [101]. The *NEIL1/NEIL2* KO showed increased locomotor activity, reduced anxiety, improved learning, and reduced levels of DNA damage in the hippocampus [102]. *NEIL1/NEIL2* and *NEIL1/NEIL2/NEIL3* KOs showed that, unexpectedly, the mice were not prone to cancer and did not show an increase in mutation frequencies under physiological conditions [103]. Although not all double knockouts have been studied, current data shows that the loss of function of multiple glycosylases has a phenotypic impact.

#### 3.1.4. Downstream BER Enzymes

Deletion of downstream BER enzymes has severe effects [76]. *APE1* KO showed embryonic lethality [104,105]. Although the heterozygous mutants appear normal, the mouse embryo fibroblasts (MEFs) and the brain cells were dying because of the sensitivity to oxidizing agents. In addition, this KO showed higher BER activity in the testis but lower activity in the liver and the brain. Furthermore, high *APE1* nuclear levels were shown to heighten the progression of ovarian carcinoma. *PCNA* KO mice were embryonically lethal [106]. *POL β* KO mice were not viable. Histological studies showed that the lungs failed [107]. Mutations found in the *POL β* gene include prostate [108,109], bladder [110], and colorectal [111] cancers. *RPA* KO in hepatocytes showed reduced expression of lipid oxidation-related genes in the epigenome [112]. In addition, fatty acid β oxidation and lipid content increased in the liver. Non-alcoholic fatty liver disease (NAFLD) caused reduced hepatic *RPA* levels in mice and humans. Although no human condition has yet been associated, *FEN1* KO showed early death during embryogenesis [113,114]. *XRCC1* KO showed embryonic lethality [115] and has been associated with human lung [116], prostate [117], colorectal [118], and breast cancer [119]. Lastly, *LIG3* KO mice embryos did not survive but are only needed for survival in mitochondrial DNA integrity, not nuclear DNA [120,121]. *LIG1* deficiency showed no repair defect, but it does increase genome instability [122].

#### 3.1.5. 5′-Gap Related Proteins

The 5′-Gap LP-BER is unique compared to the other known LP-BER pathways. Although the following studies were published before the discovery of this sub-pathway, knockouts of these genes show their importance for organism survival. *ERCC1* (*XPF*) KO showed retarded postnatal growth [123] and spontaneously developed symptoms that aligned with neurodegeneration [124]. Furthermore, hematopoietic cells undergo rapid turnover, resulting in prematurely exhausting stem cells; bone marrow progenitors are sensitive to crosslinking agents [125,126]. KO also resulted in infertility in both male and female mice [127].

Similarly, *XPF* KO showed postnatal growth defects, and the mice died approximately three weeks after birth [128]. Further studies showed the organs appeared normal but smaller, except the liver cells had enlarged nuclei. Embryonic fibroblasts were hypersensitive to UV radiation. *RECQ1* KO mice or knockdown in human cells showed increased amounts of spontaneous sister chromatid exchanges, chromosomal instability, DNA damage, and increased sensitivity to ionizing radiation [129,130].

## 4. Deficiency and Human Disease

### 4.1. Cancer

Cancer is a genetic disease caused by DNA mutations. Loss or reduced function of sequences in DNA repair genes may likely increase the mutation load in a healthy genome; this would impact tumor suppressor and oncogenes’ ability to respond to endogenous oxidative stress or environmental carcinogens. Germline single nucleotide polymorphisms (SNPs) and germline and somatic mutations in various BER genes have been associated with many human cancers. In addition, BER gene expression status is a promising prognostic and predictive biomarker in cancer.

Missense and nonsense mutations in DNA glycosylases result in hereditary syndromes such as MYH-associated polyposis (MAP) and NTH1-associated tumor syndrome (NATS). These syndromes predispose people to early adenoma, which transitions to colorectal cancers [131,132]. *D239Y* variant of *NTHL1* occurs in around 6.2% of the population; in vitro and in vivo characterization of this variant showed loss of glycosylase activity and a cancerous phenotype [133].

Multiple SNPs within the *APE1* gene are also associated with cancer. *D148E* variant is frequently associated with colorectal cancer (CRC) [134]. *L104R* and *R237C* variants showed reduced endonuclease activity and incapability for APE1’s protein–protein interactions with XRCC1 and POL β [135,136]. The clinicopathological significance of APE1 protein expression is tumor-type dependent; its post-translational modifications were correlated with patient outcomes. High *APE1* expression correlates with poor survival of osteosarcoma and non-small cell lung cancer patients (NSCLC) [137]. In contrast, low APE1 expression was significantly associated with high histological grade, high mitotic index, glandular de-differentiation, and poor survival in ER-positive breast cancer patients [138]. *APE1* sub-cellular localization also plays a role in cancer patients’ outcomes. In NSCLC patients, cytoplasmic expression of APE1 was significantly associated with poor survival rate and poor prognosis [139]; this translates for thyroid and epithelial ovarian cancer patients [140,141].

Strikingly, a mutated variant of *POL β* is present in approximately 30% of all tumors, regardless of tissue type [142]. However, *POL β* mutations are predominantly associated with human CRC [143]. Low POL β mRNA and protein expression were significantly associated with high-grade, lymph node positivity, pleomorphism, triple-negative, basal-like phenotypes, and poor patient survival in breast cancer. In contrast, in estrogen receptor-positive breast cancer patients who received tamoxifen for treatment, low POL β protein expression was significantly associated with aggressive phenotype and poor survival [144]. Epidemiological studies showed that several SNPs in XRCC1 are associated with cancer. *R194W* and *R399Q* variants are associated with breast and pancreatic cancer [119,145,146]. XRCC1 protein expression was a candidate prognostic marker and predictive factor for resectable gastric carcinoma patients who received adjuvant platinum-based chemotherapy [147]. FEN1 mRNA overexpression was also significantly associated with high-grade, ER-negative, PR-negative, triple-negative breast cancer and poor cancer-specific survival [148]. FEN1 protein expression was associated with poor survival of both ER-positive and ER-negative breast cancer [148]. Similarly, FEN1 overexpression was significantly associated with high-grade and stage and poor patient survival [148]. These findings suggest that individuals with compromised BER or aberrant BER protein expression are at risk for developing cancer.

### 4.2. Neurodegenerative

Neuronal genomes are prone to oxidative damage because high metabolic activity creates large amounts of ROS. Accumulation of oxidative genome damage and defective repair are etiologically linked to neurodegenerative diseases. Oxidative damage repair enzymes are differentially expressed in the brain compared to other organs. OGG1 expression is generally low, but PNKP, XRCC1, and LIG3 are highly expressed in the brain. Decreased OGG1 levels and mutations in the *OGG1* gene are linked to Alzheimer’s disease. OGG1 is also involved in the CAG trinucleotide repeat expansion found in Huntington’s disease.

Several neurological diseases are linked to DNA end-processing activity defects, resulting in defective gap filling and ligation failure. TDP1, APTX, and PNKP functions are required in mammalian cells to remove a discrete set of strand-break termini induced by ROS or abortive DNA metabolic intermediates. TDP1 functions to remove varieties of adducts from 3′ ends during DNA repair. Typically, it hydrolyzes the phosphodiester bond between a tyrosyl moiety and a DNA 3′-end, which has been implicated in the repair of topoisomerase 1 (TOP1)-DNA covalent complexes. Homozygous mutations in *TOP1* cause spinocerebellar ataxia with axonal neuropathy (SCAN1) [149]. APTX is involved in the resolution of stalled DNA ligation intermediates that contain adenosine monophosphate moiety covalently linked to 5′-PO_4_ termini of the strand breaks [150]. This intermediate can arise when DNA lesions, such as an abasic site at the 3′-terminus of the strand break, prevent the ligation process. *Aprataxin* (*APTX*) mutations lead to progressive ataxia-ocular motor apraxia 1 (AOA1) and peripheral neuropathy. AOA1 cells are hypersensitive to hydrogen peroxide and MMS, consistent with their role in BER/SSBR. Accumulation of DNA damage in AOA1 cells may negatively impact transcription, leading to apoptosis.

Recently, mutations in *PNKP* have been linked to microcephaly, seizures, developmental delay (MCSZ), and ataxia-ocular motor apraxia 4 (AOA4). PNKP mutations in MSCZ patients occur mainly in the phosphatase and kinase domain, causing a reduction of the protein down to 5–10% of wild-type levels; mutations linked to AOA4 are all in the kinase domain. However, none of the mutations, in either MSCZ or AOA4, abolish PNKP’s 3′-phosphatase activity; this reduced expression is sufficient for the initial stages of neurogenesis. However, the reduced PNKP level during later stages of neuronal development lead to increased DNA damage accumulation, likely in the transcriptionally active DNA, leading to apoptotic cell death and neurodegeneration [151].

### 4.3. Aging

Aging is a complex process associated with a wide variety of features at the molecular and cellular levels. Although aging is the source of most chronic diseases, little is known about its proximate causes. Among the features causally contributing to aging pathologies, DNA damage is a strong candidate for the primary cause of aging [152]. Inherited defects in DNA repair or DDR systems underlie premature aging in humans. For example, *XPF* KO in mouse models and human mutations have shown signs of premature aging [153,154,155,156].

Although endogenous DNA damage underlies significant aspects of the phenotypic signs of aging, there are strong arguments against this conclusion. One claim is that if DNA damage is the central cause of aging, then improving DNA repair should extend lifespan, but no evidence supports this correlation. DNA repair corrects various damages through well-characterized mechanisms using proteins that serve additional functions. Upregulating the activity of one or a few genes cannot ensure that DNA repair can increase longevity. Although it is not known, there is a possibility that master regulators of DNA repair affecting multiple repair systems may overcome these complexities. Another argument is that identifying and quantifying endogenous damage generated spontaneously is technically extremely difficult. This fact prevents correlating types of damage accumulation that likely impair cellular functions with age, thus contributing to age-related pathologies. However, mutations that accumulate over a defined period can now be accurately determined; this perhaps explains the increased cancer risk in old age since damage buildup is a well-known cause of cancer. Therefore, the accumulation of endogenous DNA damage is the most likely driver of aging [152].

### 4.4. Inflammation

Reactive oxygen species produced by the immune cells at the sites of infection can induce DNA damage and are repaired by BER. Besides repair functions, DNA glycosylases have been found to play additional roles, including modulation of immune response. Recently, it was shown that OGG1 has a distinct role in pro-inflammatory gene expression via modulation of nuclear factor kappa-light chain-enhancer of activated B cells (NF-κB) [157]. This is achieved by the binding of OGG1 with high affinity to its cognate 8-oxodG lesion in the guanine-rich promoter region of the pro-inflammatory genes, followed by the assembly of transcription machinery. When mice are challenged with inflammatory agents, up-regulation of pro-inflammatory genes is significantly reduced in either OGG1-deficient mice or mice treated with a small molecule non-catalytic inhibitor of OGG1, leading to considerably decreased inflammation [158,159]. These results are consistent with the observation that higher OGG1 levels may favor inflammation. Among different base modifications from oxidative stress following inflammatory stimuli, only 8-oxodG is recognized explicitly by OGG1 with high affinity. However, cytosine in GC-rich promoters is also likely to be oxidized and will induce the generation of 5-OHU, which NEIL2 preferentially recognizes. Thus, NEIL2 is a candidate for recognition of oxidized cytosine in promoters, resulting in modulation of transcription. Compared to *OGG1* KO mice, *NEIL2*-deficient mice are highly susceptible to inflammation when exposed to pro-inflammatory mediators [71]. This phenomenon is achieved through NEIL2-mediated blocking of NF-κB from binding to the promoter regions of its target genes via direct interaction with the Rel homology region of RelA and repressing pro-inflammatory gene expression. Intrapulmonary administration of purified NEIL2 significantly abrogated NF-κB binding to cognate DNA, reducing pro-inflammatory gene expression and neutrophil recruitment in mouse lungs [160]. These results are consistent with the anti-inflammatory role of NEIL2, aside from its repair functions. Therefore, NEIL2 involved in the immune response can be regulated via repair-mediated (via BER) and repair-independent pathways. Dysfunction of NEIL2 may cause an accumulation of oxidative damage and AP sites, resulting in replication fork arrest and impaired transcription, leading to the accumulation of SSBs and DSBs. The DNA fragments from these strand breaks leak into the cytoplasm and induce an inflammatory response by the cyclic GMP-AMP synthase (cGAS) pathway. Repair-independent immune regulation by NEIL2 is mediated by restricting NF-κB binding to the target promoter of an inflammatory gene, as described above.

By analyzing the publicly available transcriptomic databases of *SARS-CoV-2* infected patients, it was recently found that the level of *NEIL2* expression was inversely correlated with disease severity in COVID-19 patients [161]. Surprisingly, transcripts encoding for *NEIL2*, but not *OGG1* or *NEIL1*, were significantly decreased in the lungs of virally infected *NEIL2*-proficient mice and hamsters. Transcriptional expression and protein levels were reduced in the infected mice compared to uninfected controls. Excessive DNA damage accumulation due to decreased NEIL2 levels partially contributes to the exacerbated outcome of *SARS-CoV-2* pathogenesis [161]. Consistent with viral infections, *Helicobacter pylori* (*H. pylori*) and *Fusobacterium* (*Fn*) infection and consequently downregulation of *NEIL2* at mRNA and protein levels led to increased inflammation and oxidative damage, likely through increased production of ROS [162,163]. Together, these data strongly suggest an anti-inflammatory role of *NEIL2* in regulating the pathogenesis of viral and bacterial infection.

## 5. Medicinal Targets

Cancer cells often depend on increased BER activity to survive oxidative stress. In addition, it reduces radiotherapy and/or chemotherapy efficacy because these treatments generate cytotoxic or cytostatic base damage to cancer cells; however, enhanced BER activity repairs this damage, allowing cancer cells to continue surviving. Thus, targeting BER is considered an effective strategy to overwhelm cancer cells with DNA damage and improve the efficacy of radio- and chemotherapy. Rapidly dividing cancer cells are more prone to cell killing than most non-dividing normal cells in our body. Dividing normal cells have a lower proliferation rate compared to cancer cells. In addition, after cancer treatment, the body can recover or replenish these cells. Overwhelming base damage and SSB load in rapidly dividing cancer cells often stall DNA replication, leading to an accumulation of DSBs, which are highly lethal. Thus, inhibiting BER alone, in combination with radio- and chemotherapy, or a synthetic lethal partnership with another pathway can provide therapeutic effectiveness for cancer cells with a lesser impact on normal cells.

Small molecule inhibitors have been identified against several BER proteins for therapeutics, such as DNA glycosylases (UNG, MPG, OGG1, and NEIL1), APE1, end processing (PNKP, PARP, XPF-ERCC1, FEN1), gap-filling (POL β, POL δ/ε), and nick sealing (LIG3 and LIG1) proteins.

### 5.1. DNA Glycosylases

Small-molecule UNG inhibitors have been developed by linking the uracil substrate fragment to a library of aldehyde tethers. These molecules prevent the glycosylase from binding to the DNA strand and its glycosidic cleavage through competitive inhibition. These inhibitors are active in cell-free systems, but their potency in cancer cell lines has not been tested yet [164]. For MPG, several small molecule inhibitors (magnesium, Trp-P-1, morin hydrate (a naturally occurring flavonoid), and Aza-nucleoside (imidazol-4-ylmethylpyrrolidine)) have been developed to inhibit the glycosylase activity with reasonable potency within in vitro reactions. However, no successful in vivo results have been shown; therefore, no testing has been conducted for overcoming therapeutic resistance in glioblastoma cells [165,166,167,168]. For OGG1, several small molecule inhibitors, such as a hydrazide compound (O8) and SU0268, effectively inhibit OGG1 activity with lower IC_50_ in vitro and in vivo [169,170].

Interestingly, SU0383 acts as a dual inhibitor for OGG1 and MTH1, a nucleotide sanitizing enzyme that repairs 8-oxodG in the nucleotide pool [171]. Dual inhibition would increase the DNA oxidation load and kill cancer cells more effectively [172,173]. These OGG1 inhibitors warrant further testing in cellular and animal cancer models. TH5487 inhibits the binding of OGG1 to 8-oxodG and prevents the transcription of inflammatory response genes through the mechanisms described in Section 4.4 [158]. Several purine analogs, especially derivatives of 2-thioxanthine (2TX), have been developed as irreversible inhibitors to NEIL1 and are effective in vitro [174,175]. However, their efficacy in appropriate cancer cell and animal models must be tested.

### 5.2. AP Endonuclease (APE1)

Methoxyamine (MX or TRC102) has been used to inhibit APE1 for a long time, but it does not interfere with APE1 directly. It modifies AP sites, making them ineffective for APE1 binding. MX also inhibits the DNA lyase activity of bifunctional DNA glycosylases. MX has been used in several phase I and II clinical trials with anti-tumor drugs for different tumor types [176,177]. Few direct APE1 catalytic small molecule inhibitors have also been developed and are shown to sensitize other cancer cell lines when used in combination with chemotherapeutics, methylmethanesulfonate (MMS), and temozolomide (TMZ) [178,179,180]. APE1 also has a second activity of redox activation of specific transcription factors (e.g., NF-κB, p53, STAT3, and HIF-1α) for their DNA binding and transcription activity [181]. APE1 serves as a reducing agent, subsequently reducing the oxidized inactive transcription factors, allowing transcription to occur when needed. E3330 and Gossypol/AT101, redox inhibitors of APE1, inhibit functions of NF-κB and BCL2, respectively [182,183] and induce cytotoxicity as single agents [184] or in combination [183,185] in many cancers. Multiple clinical trials are currently investigating their efficacy.

### 5.3. End Processing Enzymes

PNKP has dual 5′-kinase and 3′-phosphatase activities on SSBs and DSBs. Imidopiperidine, especially A12B4C3, is a non-competitive inhibitor that blocks the phosphatase reaction [186,187], radiosensitizes prostate cancer cells [188], augments Auger-emitting radioimmunotherapy in human myeloid leukemia cells [189], and is an effective monotherapy in killing of cancer cells deficient in *PTEN* and *tyrosine phosphatase SHP-1* genes [190,191]. PNKP inhibitors have been delivered directly to the tumor through micelle encapsulation of the drug for radiosensitizing colon cancer cells to avoid peripheral toxicity [192].

Combining small molecule FEN1 inhibitors (NSC-281680 and SC13) and chemotherapeutics (TMZ, cisplatin, or 5-FU) sensitized colon and breast cancer cells [193,194]. Cancer cells deficient in *MRE11, CDC4, and BRCA1/2* showed higher sensitivity to these inhibitors alone [195,196]. Importantly, FEN1 expression seems to be a predictive marker for resistance to tamoxifen in *ERα*-positive breast cancers. Tamoxifen-resistant cell lines showed higher sensitivity to a novel FEN1 inhibitor (FENi#2) [197]. Since FEN1 is also required for DNA replication, the efficacy of these inhibitors in cancer cell killing targeting BER mechanisms needs to be elucidated.

Catalytic PARP inhibitors (PARPi) have been developed to inhibit PARP1, PARP2, (and PARP3) [198]. PARPi can not only disrupt the coordination of repair proteins during DNA repair but also affect chromatin accessibility and chromatin remodeling. Specificity, effectiveness of catalytical inhibition, pharmacodynamic/kinetic properties, and its ‘trapping’ ability significantly affect their efficacy in killing cancer cells [199]. These inhibitors also display different mechanisms for blocking PARP activity; talazoparib and olaparib trap PARP1/2 on DNA at the damage site [200,201] whereas veliparib causes an allosteric change in the PARP protein, thereby inducing its dissociation from DNA [199]. PARPi, as a single agent, can sensitize cancer cells that are BRCA-mutated and deficient in HR-mediated DSB repair. Several PARPi (olaparib, rucaparib, niraparib, and talazoparib) have been approved as monotherapy agents for BRCA-mutated breast, ovarian, fallopian tube, and peritoneal cancers. These inhibitors are also effective in HR-proficient cancer cells when applied with radiation [202,203,204]. Indeed, PARPi is currently used in many clinical trials for combination therapies and chemo/radiotherapy for various cancer types. Despite initial clinical success, patients often face drug resistance because of different mechanisms, including retention of HR activity in their tumor cells by secondary mutations in BRCA proteins [205]. These problems warrant better drug design and taking steps to overcome drug resistance in improving the efficacy of PARPi use in the future. Since PARPs are also involved in the repair of DSBs in addition to SSBs [206,207,208], other PARP-dependent repair mechanisms, besides BER, cannot be ruled out for PARPi-mediated cell sensitization [204,209,210]. Several small molecule inhibitors, NSC16168, NSC130813, B9, compounds 3, 4, and 6 for XPF-ERCC1 have been recently developed mainly about inhibiting its endonuclease activity or heterodimerization of ERCC1 and XPF in NER, DSBR, and interstrand cross-link (ICL) repair. These compounds also showed potential activity as chemosensitizers of many cancer drugs such as cisplatin, oxaliplatin, 5-FU, cyclophosphamide, ionizing radiation, and mitomycin C in cell culture, and, in some cases, in animal models [211,212,213,214]; they have the potential to be used to probe BER mechanisms and for testing sensitization of cancer cells to BER-relevant chemotherapeutics (MMS and TMZ) and oxidative DNA damaging agents.

### 5.4. Gap Filling Enzymes

Several small molecule inhibitors against POL β have been developed. NSC666715, designed by in silico molecular docking, and Natamycin, an antibiotic/antifungal agent, were shown to block the strand-displacement activity of POL β in LP-BER but do not specify which sub-pathway [215,216]. NSC666715 and Pro-13, an irreversible inhibitor of POL β (and POL λ), also potentiate the cytostatic and cytotoxic effects of TMZ and MMS in cancer cells [215,217]. In an alternative approach, a protein degradation pathway specific to POL β was targeted to reduce its protein level in cancer cells. Specifically, an siRNA knockdown of the deubiquitylating enzyme ubiquitin-specific protease 47 (USP47) reduced POL β protein levels and sensitized cancer cells to MMS and hydrogen peroxide [218]. Aphidicolin is a potent inhibitor for POL δ/ε and POL α. Aphidicolin has been shown to inhibit LP-BER and increase the sensitivity of cancer cells to MMS [219,220,221]. However, these POLs are essential for DNA replication and recombination. Thus, cell sensitivity cannot be attributed to BER only.

### 5.5. Nick Sealing Enzymes

Available DNA LIG1 and three structures were exploited to design a series of inhibitors against them [222,223,224]. Inhibitors that targeted ligase(s) were cytostatic or cytotoxic to cancer cells and increased the sensitivity of cancer cells to MMS or ionizing radiation but did not have the same impact on normal cells, highlighting its application in cancer therapy [225]. However, like FEN1, ligases are also needed for replication and additional cellular pathways.

## 6. Conclusions and Future Perspectives

Base excision repair is a complex repair system with various sub-pathways, has biological relevance in maintaining genome stability and preventing disease, and provides potential targets for therapeutics. Although the pathways are well established from a mechanism standpoint, a few questions warrant further studies. LP-BER can carry out a 2–11 nucleotide gap repair, but no studies explain how the patch size is regulated. In addition, within the Hit and Run and Polymerase switch sub-pathways, there is one identical step—one new nucleotide is incorporated with a nick site and 5′-modified dRP remaining. However, how do the cells decide to proceed with FEN1 (for Hit and Run) or POL δ/ε (for Polymerase Switch)? Lastly, for the Polymerase Switch, Non-Polymerase Switch, and 5′-Gap sub-pathways, after FEN1 cleaves the flap, what prevents POL δ/ε from returning to the nick site before ligation since it initially enters the pathway at a nick site?

The newest sub-pathway, 5′-Gap, leaves room for research related to its biological context. A working theory is that this sub-pathway can remove 3′ blocks. PNKP removes 3′-PO_4_ groups; however, mutations in this gene can lead to severe neurological diseases and cause seizures [226]. Although individuals with PNKP mutations are more sensitive to DNA-damaging agents, no case of cancer or immunodeficiency has been reported. Global PNKP KO/KI mice were shown to be embryonically lethal [227]. Since PNKP is the only known protein to remove 3′ blocks, the 5′-Gap pathway could serve as an alternate system for individuals with its mutations or deficiencies.

In addition, the role of PARP1 is not fully understood in the context of this sub-pathway. It has been shown that PARP1 is needed, but not its activity [15]. Rather than being triggered by an oxidized or reduced 5′-dRP (common in other LP-BER sub-pathways), 8-oxodG incorporation could activate the 5′-Gap pathway. 8-oxodG can generate a 3′ block, which can be removed during DNA unwinding by RECQ1. 8-oxodG misincorporation can be detrimental because POL β can continue to repair or create deformed structures when bound to adenine using a Hoogsteen edge in the syn conformation [228]. In the anti-conformation, 8-oxodG binds as if it is an unoxidized base. This publication contrasts previous work, which showed that 8-oxodG only pairs as a Watson–Crick base pair [229]. Regardless of conformation, 8-oxodG has almost no effect on the three-dimensional structure [230]. However, the base can be further oxidized, which has biological consequences. Downstream effects would lead to ligation failure [28], which could be solved in an error-free manner by the 5′-Gap sub-pathway.

Lastly, the mouse models for *RECQ1* and *XPF* KOs showed the potential functions of these genes related to BER, even though these studies preceded the discovery of this sub-pathway. BER defects lead to SSB accumulation, translating to DSB accumulation and cell death. Researchers showed low survivability in mice with either knockout, which supports that *RECQ1* and *XPF* could be needed in both BER and DSBR to protect cells from endogenous DNA damage because if they were not involved in BER, then the absence of these genes would not affect mice survival. *XPF* KOs in mouse models and humans with *XPF* mutation have shown signs of premature aging. However, this work was conducted in the context of referencing XPF as an NER protein. Although it has been established that XPF is involved in BER through the 5′-Gap sub-pathway, there is no direct correlation between XPF, BER, and premature aging. RNAPIIo ubiquitination has recently been shown to trigger TC-NER of UV-induced damage [231]. A similar mechanism for initiating transcription-associated BER of oxidative damage remains to be elucidated.

Aside from providing genome stability, BER is essential in preventing human diseases, specifically inflammation. Evidence shows inflammation is a risk factor in the development of cardiovascular diseases [232]. However, according to the American Heart Association, understanding this correlation needs further work [233]. Furthermore, obesity predisposes individuals to inflammatory issues, which was studied in the context of understanding inflammatory factors [234]. There is a connection between BER and inflammation; since inflammation has also been linked to cardiovascular disease and obesity, potential correlations between BER and obesity must be studied to discover novel therapeutics. In addition to inflammation, the connection between BER genes and cancer is being studied, but only for a few proteins. RECQ1 and XPF have been recently discovered in the context of BER because they are unique to the 5′-Gap sub-pathway; it is crucial to understand the relationship between these genes in the context of BER and its potential effect on cancer.

Although there are various BER proteins, only a fraction has been considered as inhibitors for therapeutics. Ongoing work would need to include considering other proteins. In addition, current inhibitors are not ready for clinical trials for various reasons; finding solutions will allow for these inhibitors to move through the drug pipeline. Specifically, for APE1, inhibitors being currently tested target its redox function, but not when it acts as a coactivator. Overall, studying BER in the context of medicinal targets is an ongoing area of research.

## Data Availability

Not applicable.
